# Evaluation of standard diagnostic rapid test kits for malaria diagnosis among HIV patients in Kano, Nigeria

**DOI:** 10.4102/ajlm.v7i1.698

**Published:** 2018-12-05

**Authors:** Henry A. Mbah, Feyisayo E. Jegede, Surajudeen A. Abdulrahman, Tinuade I. Oyeyi

**Affiliations:** 1 LabTrail Global LLC, Smyrna, Delaware, United States; 2 Biological Science Department, Bayero University Kano, Gwarzo Road Kano, Nigeria; 3 Family Health International-360, Garki,Federal Capital Territory, Abuja, Nigeria; 4 Department of Public Health, Penang Medical College, Penang, Malaysia

## Abstract

**Background:**

Malaria diagnosis among HIV-positive patients is uncommon in Nigeria despite the high burden of both diseases.

**Objectives:**

We evaluated the performance of a malaria rapid diagnostic test (MRDT) against blood smear microscopy (BSM) among HIV-positive patients in relation to anti-retroviral treatment (ART) status, CD4+ count, fever, cotrimoxazole prophylaxis and malaria density count.

**Method:**

A cross-sectional study involving 1521 consenting randomly selected HIV-positive adults attending two ART clinics in Kano, Nigeria, between June 2015 and May 2016. Venous blood samples were collected for testing with MRDT, BSM, and CD4+ T cells count by cytometry. Biodata and other clinical details were extracted from patient folders into an Excel file, cleaned, validated, and exported for analysis into SPSS version 23.0. Sensitivity, specificity, predictive values of MRDT were compared with BSM with a 95% confidence interval.

**Results:**

Malaria parasites were detected in 25.4% of enrollees by BSM and 16.4% by MRDT. Overall sensitivity of MRDT was 58% and specificity was 97%. Cotrimoxazole prophylaxis and fever status did not affect MRDT sensitivity and specificity. Unexpectedly, the sensitivity was highest at parasite density count of less than 500 cells/µL. At CD4+ T cells count over 500 cells/µL the sensitivity was higher (62.4%) compared to 56% at less than 500 cells/µL. In the non-ART group sensitivity was higher (65%) compared to those on ART (56%) but the specificity was similar. All differences were significant for all variables (*p* < 0.05).

**Conclusion:**

Although the MRDT specificity was good, the sensitivity was poor, requiring further evaluation for use in malaria diagnosis among HIV-malaria co-infected persons in these settings.

## Introduction

Malaria remains a major public health problem in Nigeria with 97% of the population at risk.^1^ In 2016 Nigeria had about 57.3 million cases of malaria with over 112 000 deaths reported.^2^ Although malaria prevalence in Nigeria varied between states, a 60.6% prevalence has been reported in a population-based study across five local government areas of Kano State.^3^ HIV prevalence among adults in Nigeria was about 2.9% with about 3.2 million persons living with HIV in 2016.^4^ The greatest burden of disease due to both HIV and malaria (predominantly *Plasmodium falciparum*) occurs commonly in sub-Saharan Africa^5^ and the rates of co-infection make interaction between both diseases a global health focus.^5,6,7^

A key factor for effective management of malaria is early and accurate diagnosis. The global impact of malaria has prompted an increase in the development of diagnostic strategies. The updated World Health Organization (WHO) recommendations indicate that all suspected cases of malaria must be confirmed with a laboratory diagnostic test before treatment.^8^ This enables differentiation of malarial and non-malarial fevers, to prevent unnecessary use of antimalarial drugs.^9,10^ This is very crucial among HIV-positive patients, where febrile illnesses other than malaria are common. Opportunistic infections and other febrile illnesses mimic malaria in HIV patients.^5^

Although microscopy is the current gold standard for the diagnosis of malaria, it is time consuming and labour intensive.^11,12,13^ In many malaria-endemic areas like Nigeria, it is difficult to maintain the required technical skills, equipment and infrastructure particularly in rural areas,^14,15^ where the disease is more prevalent. Hence, the use of rapid diagnostic tests (RDT) in such endemic areas is recommended.^16,17^ Furthermore, part of the national malaria control strategy plan for 2014 to 2020, with a vision of having a malaria-free Nigeria, is to test all care-seeking persons suspected of suffering from malaria using RDT or microscopy.^1^

Data from reviewed literature showed that very few studies have evaluated the validity of RDT for malaria diagnosis in the general population in Nigeria.^14,15,18^ Thus far, only one study has evaluated the performance of a malaria RDT (MRDT) among 387 HIV-positive persons in Nigeria.^19^ Overall MRDT sensitivity, specificity, positive and negative predictive value have not yet been properly evaluated especially among HIV-positive individuals.^20,21^

The objective of the study was to evaluate the performance of a commercially available MRDT (based on *P. falciparum* histidine-rich protein type-2 and *Plasmodium* lactate dehydrogenase), the Standard Diagnostics (SD) Bioline MRDT, compared to the gold standard, microscopy, in the diagnosis of malaria among HIV-positive participants with respect to malaria parasite density, CD4+ T cells count, cotrimoxazole prophylaxis use, anti-retroviral treatment (ART) status and occurrence of fever. The choice of SD Bioline was on the premise that this rapid test kit is the most commonly used MRDT for routine malaria diagnosis in Kano State.

## Materials and methods

### Ethical considerations

The protocol was reviewed by Kano State Hospitals Management Board local ethical committee and written approval was given (study approval number: HMB/GEN/488/1 of 17/04/2015). Participant consent was obtained from all respondents.

### Study setting and population

This was a cross-sectional study carried out from June 2015 to May 2016 at two health facilities in Kano City, Nigeria, a malaria endemic area.^3^ The facilities are Infectious Diseases Hospital Kano (IDH) and Murtala Mohammed Specialist Hospital Kano.

The population of interest in this study were HIV-positive adult participants who routinely visit the two health facilities to access ART services. They consisted of male and female (non-pregnant) patients (on ART and non-ART) aged 18 years and older enrolled in an ART programme who had not been on an antimalarial drug in the past 14 days and who willfully consented to participate in the study.

### Data collection and sampling technique

A total of 1521 consenting participants were randomly selected and blood specimens were collected and analysed for the presence of malaria parasite using both RDT and microscopy techniques and density of malaria parasites was determined. Additionally, relevant socio-demographic and clinical data including clients’ code, age, gender, use of co-trimoxazole prophylaxis, ART status (receiving or not receiving treatment), and episodes of fever were retrieved from both patients’ folders and Lafiya Management Information System (LAMIS^®^) software with the support of trained data entry clerks. We ensured data confidentiality through participant coding and restricting access to the data to only research team members.

### Laboratory analysis (rapid diagnostic test, blood microscopy and parasite density determination)

From 1521 consenting HIV-positive participants, 4 mL venous blood sample was aseptically collected using sterile vacutainer needle and holder into an ethylenediaminetetraacetic acid (EDTA) anticoagulant tube. The blood samples were mixed properly to avoid clotting before laboratory investigations.

We used the MRDT kit (Code 05FK30) as described by the manufacturers (Standard Diagnostics Bioline Korea, 2013).

For microscopy slide method, both thin and thick smear slides were prepared and examined. The microscopy procedure adopted was described by WHO.^23^ To ensure the quality of testing, MRTD and microscopy were performed by independent laboratory personnel and MRTD results were blinded to microscopists. All MRTD kits were stored according to manufacturer recommendation and used before the expiry date. The two microscopists responsible for slide examinations in both facilities have attended WHO training on malaria microscopy. Additionally, 10% of the slides were randomly selected for re-testing. About 2% of the results were discordant between the first and second readers and were subjected to further evaluation by a third reader before a final decision was made.

Malaria parasite density for each positive smear was calculated using individual white blood cell count according to this formula:

$$\begin{array}{l} \text{malaria parasite count}=\text{Number of parasite out}\times \\ \;\text{patienta actual white blood cell}\\ \;\text{count}/\mu \text{L}/\text{Number of white}\\ \;\text{blood cell count}200\text{ or }500. \end{array}$$

### Data processing and analysis

Data were reviewed, cleaned and validated in a Microsoft 2013 Excel file (Microsoft Corp., Redmond, Washington, United States) and analysed using Statistical Package for Social Scientists (SPSS version 23.0; IBM, Armonk, New York, United States). The sensitivity, specificity, positive predictive value and negative predictive value of MRDT were estimated against blood smear microscopy (BSM), the gold standard, at 95% confidence interval in relation to (1) malaria parasite density count, (2) fever (presence or absence), (3) CD4+ T cells count, (4) co-trimoxazole prophylaxis use, and (5) ART status (currently on ART or not taking ART). In addition, differences in performance of MRDT against BSM were analysed (overall and for sub-groups of the five variables mentioned above) based on level of ‘equality of marginal positives’ in disease classification between the two using McNemar tests and significance level was set at *p* < 0.05.

## Results

### Study population

This study included a total of 1521 HIV-positive participants consisting of 1074 (70.6%) women and 447 (29.4%) men. The mean age was 37.20 with a standard deviation (SD) of 10.41 years.

Data on clinical information showed that the majority (84.7%) were on ART. An almost equal proportion of the participants (*n* = 772, 50.8%) were on daily dose co-trimoxazole prophylaxis for prevention against opportunistic infection including malaria parasite infection while 749 (49.2%) were not on co-trimoxazole prophylaxis. About (7%) complained of fever. The mean current CD4+T cells count of the participants was 401.35 cells/µL ± 239.24 cells/µL, and ranged from 6 cells/µL to 1736 cells/µL. The majority (*n* = 1038, 68.2%) of the respondents had a CD4+T cells count lower than 500 cells/µL, while 31.8% (*n* = 483) had CD4+ T cell counts of 500 cells/µL or higher. The mean malaria parasite density was 265 cells/µL ± 31.8 (SD) cells/µL with a range of 20 cells/µL to 2500 cells/µL. A majority (87.8%) had malaria parasite density less than 500 cells/µL, while the rest (12.2%) had parasite density 500 cells/µL or higher.

### Prevalence of malaria parasite among HIV-positive patients based on microscopy versus malaria rapid diagnosis test

The prevalence of malaria was 25.4% with BSM technique (gold standard method) and only 16.4% with MRDT. It is worth nothing that participants were recruited at routine ART follow-up visits, with or without symptoms of malaria. Almost all (99.2%) malaria infections were *Plasmodium falciparum* species while dual infection of *Plasmodium falciparum* and *Plasmodium vivax* species occurred in only three (0.8%) participants (Table 1). The overall sensitivity of the MRDT method was 58% and specificity 97%. The positive predictive value was 89% and the negative predictive value was 87% (Table 2). A 2.5% (28/1135) false positive result and 27.6% (55/200) false negative result were observed.

**TABLE 1:** Prevalence of malaria parasite among HIV-positive patients determined by malaria rapid diagnosis test and standard blood smear microscopy.

Variables	Malaria positive *n*	Percentage	Malaria negative *n*	Percentage
RDT MP parasite species *Pf&Pv*	250	16.4	1271	83.7
Prevalence of malaria & HIV	386	25.4	1135	74.6
BSM MP parasite specie *Pf*	383	25.2	–	-
BSM MP parasite species *Pf&Pv*	3	0.2	–	-
Overall *Pf* species prevalence	383	99.2	–	-
Mixed infection prevalence	3	0.8	–	-

BSM, blood smear microscopy significant difference; MP, Malaria parasites; *n*, number; *Pf, Plasmodium falciparum*; *Pv, Plasmodium vivax*; HIV, human immunodeficiency virus; RDT, rapid diagnostic test.

**TABLE 2:** Overall performance of malaria rapid diagnosis test in comparison with standard blood smear microscopy among HIV positive participants.

RDT result	Standard BSM								
	Positive	Negative	Sensitivity	Specificity	PPV	NPV	LR +ve	LR -ve	McNemar test (P value)
Positive	222	28	57.5 (52.41–62.50)	97.3 (96.45–98.35)	88.8 (84.48–92.03)	87.1 (85.73–88.35)	21.29	0.44	< 0.001
Negative	164	1107	-	-	-	-	-	-	-

**Total**	**386**	**1135**	**-**	**-**	**-**	**-**	**-**	**-**	**-**

HIV, human immunodeficiency virus; BSM, blood smear microscopy; LR, likelihood ratio; NPV, negative predictive value; PPV, predictive positive value, RDT, rapid diagnostic test.

Overall, there was significant difference in MRDT performance compared to BSM (*p* < 0.001), with the MRDT demonstrating a ‘fair’ performance and discriminatory capacity of 0.775 (Area under the curve = 0.775, 95% CI: 0.743–0.804) of the studied population.

### Performance of malaria rapid diagnostic test in comparison with standard microscopy

#### Different malaria parasite densities

The MRDT showed a sensitivity of about 72% among HIV-positive patients with malaria density count between 0 and 499 cells/µL and greater than 1000 cells/µL. Unexpectedly, a low sensitivity of about 34% was observed when density was between 500 and 999 cells/µL (Table 3).

**TABLE 3:** Performance of malaria rapid diagnostic test in comparison with standard blood smear microscopy at different malaria parasite density, fever status, cotrimoxazole prophalaxis,CD4+ count and ART status

RDT result	Standard BSM						
	Positive	Negative	Sensitivity (95% CI)	Specificity (95% CI)	PPV (95% CI)	NPV (95% CI)	McNemar test (*P*)
**Parasite density 0–499** ***µ*****L**							
Positive	145	28	72.5 (65.76–78.56)	97.5 (96.45–98.35)	83.8 (78.06–88.29)	95.3 (94.14–96.18)	0.004
Negative	55	1107	-	-	-	-	-
**Total**	**200**	**1135**	-	-	-	-	-
***Parasite density 500–999 µL***							
Positive	50	0	33.8	NA	100.0	NA	< 0.001
Negative	98	0	-	-	-	-	-
**Total**	**148**	**0**	-	-	-	-	
***Parasite density ≥ 1000 µL***							
Positive	27	0	71.1	NA	100.0	NA	< 0.001
Negative	11	0	-	-	-	-	-
**Total**	**38**	**0**	-	-	-	-	-
**Fever**							
Positive	23	1	54.8 (39.72–69.84)	98.4 (95.31–99.99)	95.8 (87.83–99.97)	76.5 (67.34–85.81)	< 0.001
Negative	19	62	-	-	-	-	-
**Total**	**42**	**63**	-	-	-	-	-
**No Fever**							
Positive	199	27	57.8 (52.63–63.14)	97.5 (96.53–98.42)	88.1 (83.80–92.32)	87.8 (86.04–89.72)	< 0.001
Negative	145	1045	-	-	-	-	-
**Total**	**344**	**1072**	-	-	-	-	-
**On CTX**							
Positive	97	18	57.7 (49.89–65.31)	97.0 (95.33–98.22)	84.3 (77.05–89.64)	89.2 (87.36–90.79)	< 0.001
Negative	71	586	-	-	-	-	-
**Total**	**168**	**604**	-	-	-	-	-
**Not on CTX**							
Positive	125	10	57.3 (50.48–63.99)	98.1 (96.56–99.09)	92.6 (87.00–95.89)	84.9 (82.76–86.73)	< 0.001
Negative	93	521	-	-	-	-	-
**Total**	**218**	**531**	-	-	-	-	-
***CD4+ count ≤ 500 cells/µL***							
Positive	164	22	56.0 (50.08–61.74)	97.0 (95.56–98.14)	88.2 (82.99–91.93)	84.9 (83.12–86.45)	< 0.001
Negative	129	723	-	-	-	-	-
**Total**	**293**	**745**	-	-	-	-	-
***CD4+ count > 500 cells/µL***							
Positive	58	6	62.4 (51.72–72.21)	98.5 (96.68–99.43)	90.6 (81.14–95.60)	91.6 (89.41–93.45)	< 0.001
Negative	35	384	-	-	-	-	-
**Total**	**93**	**390**	-	-	-	-	-
**On ART**							
Positive	178	27	56.0 (50.33–61.51)	97.2 (95.98–98.16)	86.8 (81.78–90.64)	87.1 (85.61–88.41)	< 0.001
Negative	140	943	-	-	-	-	-
**Total**	**318**	**970**	-	-	-	-	-
**Non-ART**							
Positive	44	1	64.7 (52.17–75.92)	99.4 (96.67–99.98)	97.8 (86.08–99.68)	87.2 (83.20–90.41)	< 0.001
Negative	24	164	-	-	-	-	-
**Total**	**68**	**165**	-	-	-	-	-

BSM, blood smear microscopy; CI, confidence interval; CTX, cotrimoxazole; RTD, rapid diagnostic test; ART, antiretroviral therapy; NA, Not available; LR, likelihood ratio; NPV, negative predictive value; PPV, predictive positive value.

MRDT performance was significantly different from BSM findings, regardless of malaria parasite density count of participants (*p* < 0.005).

#### Presence or absence of fever

The MRDT showed slightly lower but similar sensitivity in cases with and without fever, but similar specificity for both groups of participants. However, positive predictive value was higher (95.6%) among clients that complained of fever compared to those without (88.1%) fever (Table 3). In those with or without fever, MRDT performance was significantly lower compared to BSM findings (*p* < 0.0)

#### Co-trimoxazole prophylaxis

MRDT performed similarly in sensitivity and specificity in both groups, although higher positive predictive value was observed among participants who were not on co-trimoxazole (Table 3). Regardless of participant’s co-trimoxazole status, the performance of MRDT was significantly lower in sensitivity and specificity compared to BSM findings (*p* < 0.001) in both groups.

#### Current CD4+T cells count/µL

MRDT revealed higher (sensitivity 62%, specificity 99%, positive predictive value 91% and negative predictive value 91%) diagnostic accuracy among participants with CD4+T cells count greater than 500 cells/µL. This was lower (sensitivity 56%, specificity 97%, positive predictive value 88% and negative predictive value 85%) among participants with CD4+T cells count under 500 cells/µL (Table 3). The observed differences in performance were statistically significant (*p* < 0.001).

#### Anti-retroviral treatment status

The MRDT revealed a higher (65%) sensitivity among HIV participants who were not on ART compared (56%) to those on ART. A similar pattern of higher positive predictive value (98%) was observed among the non-ART group compared to 87% among the ART group (Table 3). For both groups of participants (ART vs non-ART), the observed differences in performance of MRDT were significantly lower in sensitivity compared to BSM findings among the non-ART group (*p* < 0.001).

## Discussion

We detected malaria parasites in 25.4% of participants by BSM and 16.4% by MRDT. Overall sensitivity of MRDT was 58% and specificity was 97%. The sensitivity and specificity were similar irrespective of co-trimoxazole and fever status. At malaria parasite density count of under 500 cells/µL, sensitivity was 73% and between 500 and 999 cells/µL, sensitivity was 34%. At CD4+ T cell count over 500 cells/µL the sensitivity was higher (62.4%) compared to 56% at under 500 cells/µL. In the non-ART group sensitivity was higher (65%) compared to those on ART (56%) but the specificity was similar.

Although blood smear microcopy still remains the gold standard for malaria diagnosis, several limitations ranging from infrastructure and equipment to competence still exist in endemic areas like Nigeria. These justify the need for an alternate method like MRDT.^15^ Since WHO recommended the use of MRDT, several test kits have flooded the market, hence the need for constant field validation to verify manufacturers’ claims. This is critical, particularly among HIV-positive patients in whom other febrile illnesses and infections mimic malaria.^5^

The prevalence of malaria in HIV-positive patients in this study was 25.4% based on microscopy compared to 16.4% based on MRDT method. This was similar to a Tanzanian study reporting a malaria prevalence of 23.8% using microscopy method among HIV-positive patients compared to 17.5% with MRTD^24^. However, our findings varied from a similar study in Lagos, Nigeria, involving a smaller sample size of 387 HIV patients where a malaria prevalence of about 19% was reported for both BSM and MRDT of another brand^19^. Another study in Burkina Faso among 114 HIV-positive participants showed a malaria prevalence of about 45% for MRDT and 42% for BSM.^21^ It is worth noting that in this study, participants were recruited at routine follow-up visits, regardless of suspicion of malaria unlike the other studies comparing performance of BSM and MRDT in HIV-positive patients suspected of suffering from or having symptoms of malaria. For example, the Tanzania^24^ and Burkina Faso^21^ studies looked at HIV-positive children suspected of malaria, while those from Lagos,^19^ Uganda,^10,20^ and Malawi^25^ looked at adults with HIV attending outpatient clinics suspected of having malaria. Thus, the direct comparison of proportions positive for malaria in our studies with the others must be considered with caution.

MRDT overall sensitivity and specificity reported in this study was similar to a 55.4% sensitivity reported in Lagos, Nigeria, but varied in specificity (90.3%).^19^ Contrary to our results, a 100% sensitivity and similar specificity of about 95.4% was documented in a Burkina Faso study among HIV-positive participants.^21^ Other studies on MRDT performance among HIV-positive population in Malawi,^25^ and two different studies in Uganda^10,20^ have shown higher sensitivity of over 94%. In these studies, the specificities averaged over 97% like in ours except for the Malawi study that reported about 51% specificity.^25^

The 2.5% false positive results observed with the use of MRDT may be an indicator of residual antigenemia. The average time that histidine rich protein-2 (pFHRP-2) remains positive after resolution of parasitaemia is about 2 weeks, although it has been shown that the protein can take over a month to clear.^26^ Using MRDT, a false positivity rate of 5.9% had been reported in Burkina Faso among HIV-positive patients.

The 27.55% false negative results observed might be partly due to deletion or mutation of HRP2 gene in the malaria parasite, which is the most common target for MRDT.^27,28^ It has been reported to exhibit a high level of polymorphism.^29,30^ This is an important factor that may affect the performance of MRDT based on antigen detection.^31,32^ In addition, false negative results have been associated with blood samples taken beyond the period of malaria fever paroxysms.^33^ *P. falciparum* isolates without the HRP2 gene have been shown to be important contributors to false negative HRP2-based MRDT testing.^34,35^ Moreover, only *Plasmodium falciparum* releases HRP2; therefore, the presence of non-falciparum infection as another malaria species co-infection in *Plasmodium falciparum* may also give a false negative result. Other factors that might possibly affect MRDT field performance are shelf life and heat stability.^36,37^ In this study, the test kits were stored based on manufacturers’ recommendations and used before the expiry date on the MRDT kits.

Unexpectedly, with the MRDT, we observed a lower sensitivity of 34% among HIV-positive participants with a malaria density count between 500 cells/µL and 999 cells/µL compared to a 73% sensitivity when the malaria density count was less than 500 cells/µL. However, the specificity at between 500 cells/µL and 999 cells/µL was 97.5%. This was contrary to a previous study in Lagos, Nigeria, that observed an increase in sensitivity from 90.9% at parasite densities greater than 200 cells/µL to 97.6% at densities greater than 500 cells/µL.^19^ Generally, at lower parasitaemia, variability in sensitivity is more common.^20,38,39^ Similar observation of false negative MRDT results at parasite counts even higher than 1000 cells/µL have been reported in other studies^19,25,40,41^ and may be attributed to prozone effect observed with immunochromatographic tests such as MRDT.^42^ All the same, many MRDTs today can achieve excellent sensitivity and specificity for *P. falciparum* at parasite density lower than 500 cells/µL.^11^

Besides this study, and to the best of our knowledge, only one other study has assessed performance of MRDT among HIV-positive patients based on febrile status in Africa.^10^ MRDT performance in our study showed similar sensitivity and specificity as reported by Mills *et al*.,^10^ in febrile or non-febrile cases in rural Uganda. Apparently, MRDT reliability is independent of febrile status.

MRDT performance was similar in terms of sensitivity, specificity and positive predictive value regardless of whether a participant was on co-trimoxazole prophylaxis or not. Information on similar studies is not available; however, with microscopy a relatively higher prevalence has been reported on those without co-trimoxazole prophylaxis compared with those on it.^19,22,43^

MRDT performance revealed a higher sensitivity among participants with a CD4+ T cell count greater than 500 cells/µL compared to a lower sensitivity among participants with a CD4+ T cell count under 500 cells/µL. Although information on similar studies is not available, it has been reported in a similar setting that HIV-positive participants with CD4+ T cell counts 250 cells/µL or higher are significantly less likely to have patent parasitaemia.^19^

The MRDT revealed a higher sensitivity among HIV-positive participants who were non-ART compared to those on ART. Lower incidence of malaria among HIV-positive adults on ART have been reported in different settings^10,43,44^ as well as reports of antimalarial properties of some antiretroviral drugs.^45,46^

Based on other reports in the general population, the use of MRDT method as an alternative to BSM in malaria endemic areas is recommended for epidemiological studies. However, the performance varied depending on species of the malaria parasite, level of parasitaemia, and immunity.^47^ For some of these reasons, such recommendation must be taken with caution in HIV-malaria co-infected persons, even though no differential effect of HIV infection on MRDT performance (specifically the SD Bioline antigen-based method) has been reported in literature.^25^

### Strengths and limitations

This study was sufficiently powered having a large sample size of 1521 HIV-positive participants across the two health facilities. Apparently, this may be the first study to assess the performance of MRDT in an African setting of significantly high HIV and malaria co-morbidity in association with variables like immune status, parasitaemia and some clinical (co-trimoxazole prophylaxis, fever and ART status) factors.

The cross-sectional design of this study was a limitation as it was difficult to determine how the potential antimalarial effect of co-trimoxazole prophylaxis and ART could have affected MRDT performance over time. Note that we observed better diagnostic accuracy of MRDT among participants who were neither on co-trimoxazole prophylaxis nor on ART. Secondly, we did not measure individual body temperature to correlate it with participants’ reports of fever.

### Conclusion

As observed in this study, the sensitivity of SD Bioline, which is based on *P. falciparum* histidine-rich protein type-2 and *Plasmodium* lactate dehydrogenase, is lower than WHO recommendation for MRDT.^48^ Thus, further evaluation is required to determine its suitability in malaria diagnosis among HIV-malaria co-infected patients in these settings.

It is worth speculating that a similar observation with the MRDT could have been observed in non–HIV-positive persons.

### Trustworthiness, Reliability and Validity

The data reported in this current article reflect findings of work done on evaluation of standard diagnostic Bioline rapid test kits for malaria diagnosis among HIV patients in Kano, Nigeria, by our research team members who all participated in the study design, execution, collection, analysis of data and report writing.

The research outcomes presented in this report were from the diagnostic evaluation of standard diagnostic Bioline rapid test kits for malaria diagnosis among HIV-positive participants in Kano, Nigeria, at Infectious Diseases Hospital and Murtala Mohammed Specialist Hospital Kano. Although previous studies have reported on the evaluation of rapid malaria diagnosis against gold standard microscopy methods with a focus on the general population, it is worth noting that data among HIV-positive persons under different conditions are not readily available. Hence, this study sought to address paucity of data on performance evaluation of standard diagnostic Bioline among HIV-positive patients in relation to various factors such as fever status, CD4+ cell count, ART status, co-trimoxazole prophylaxis and malaria density.

The outcomes of this study and proposed recommendations could be useful in public health programme planning in such settings where HIV and malaria burden is high. Apart from disease prevalence data reported, malaria density count and performance of rapid test kits was also evaluated in addition to other clinical and useful variables. This may serve as a baseline upon which comparison may be made in future.

## References

[1] United States Agency for International Development President’s Malaria Initiative Nigeria: Malaria operational plan FY 2013 [homepage on the Internet]. President’s Malaria Initiative; 2015 p. 1–74. Available from: https://www.pmi.gov/docs/default-source/default-document-library/malaria-operational-plans/fy-15/fy-2015-nigeria-malaria-operational-plan.pdf?sfvrsn=6

[2] World Health Organization World malaria report 2017 [homepage on the Internet]. 2017 Available from: http://apps.who.int/iris/bitstream/10665/259492/1/9789241565523-eng.pdf?ua=1

[3] Dawaki S, Al-Mekhlafi HM, Ithoi I, et al Is Nigeria winning the battle against malaria? Prevalence, risk factors and KAP assessment among Hausa communities in Kano State. Malar J [serial online]. 2016;15:351 Available from: http://www.ncbi.nlm.nih.gov/pubmed/27392040 10.1186/s12936-016-1394-3 PMC4938925 27392040

[4] AVERT.org HIV and AIDS in Nigeria [homepage on the Internet]. 2017 Available from: https://www.avert.org/professionals/hiv-around-world/sub-saharan--africa/nigeria

[5] Cunnington A, Riley EM HIV and malaria co-infection Immunity to parasitic infection. 2012, p. 335–352. Hoboken, NJ: John Wiley & Sons https://doi.org/10.1002/9781118393321.ch19

[6] Abu-Raddad LJ, Patnaik P, Kublin JG Dual infection with HIV and malaria fuels the spread of both diseases in sub-Saharan Africa. Science [serial online]. 2006;314(5805):1603–1606. Available from: http://www.ncbi.nlm.nih.gov/pubmed/17158329 10.1126/science.1132338 17158329

[7] Cohen C, Karstaedt A, Frean J, et al Increased prevalence of severe malaria in HIV-infected adults in South Africa. Clin Infect Dis [serial online]. 2005;41(11):1631–1637. Available from: http://www.ncbi.nlm.nih.gov/pubmed/16267737%5Cnhttp://cid.oxfordjournals.org/content/41/11/1631.full.pdf 10.1086/498023 16267737

[8] World Health Organization Guidelines for the treatment of malaria [homepage on the Internet]. 2nd ed. WHO; 2010, 197 p. Available from: http://whqlibdoc.who.int/publications/2010/9789241547925_eng.pdf

[9] Batwala V, Magnussen P, Nuwaha F Comparative feasibility of implementing rapid diagnostic test and microscopy for parasitological diagnosis of malaria in Uganda. Malar J [serial online]. 2011;10:373 Available from: http://www.pubmedcentral.nih.gov/articlerender.fcgi?artid=3269399&tool=pmcentrez&rendertype=abstract 10.1186/1475-2875-10-373 PMC3269399 22182758

[10] Mills LA, Kagaayi J, Nakigozi G, et al Short report: Utility of a point-of-care malaria rapid diagnostic test for excluding malaria as the cause of fever among HIV-positive adults in rural Rakai, Uganda. Am J Trop Med Hyg. 2010;82(1):145–147. https://doi.org/10.4269/ajtmh.2010.09-0408 20065011 10.4269/ajtmh.2010.09-0408 PMC2803525

[11] Wongsrichanalai C, Barcus MJ, Muth S, et al A review of malaria diagnostic tools: Microscopy and rapid diagnostic test (RDT). Am J Trop Med Hyg. 2007;77(Suppl. 6):119–127. https://doi.org/10.3126/ajms.v1i2.2965 18165483

[12] Hamer DH, Ndhlovu M, Zurovac D, et al Improved diagnostic testing and malaria treatment practices in Zambia. JAMA [serial online]. 2007;297(20):2227–2231. Available from: http://eutils.ncbi.nlm.nih.gov/entrez/eutils/elink.fcgi?dbfrom=pubmed&id=17519412&retmode=ref&cmd=prlinks%5Cnpapers2://publication/doi/10.1001/jama.297.20.2227 10.1001/jama.297.20.2227 PMC2674546 17519412

[13] Bell D, Peeling RW, WHO-Regional Office for the Western Pacific/TDR Evaluation of rapid diagnostic tests: Malaria. Nat Rev Microbiol. 2006;4:34–38. https://doi.org/10.1038/Nrmico1524 10.1038/nrmicro1524 17034070

[14] Uzochukwu BSC, Obikeze EN, Onwujekwe OE, et al Cost-effectiveness analysis of rapid diagnostic test, microscopy and syndromic approach in the diagnosis of malaria in Nigeria: Implications for scaling-up deployment of ACT. Malar J. 2009;8:265 https://doi.org/10.1186/1475-2875-8-265 19930666 10.1186/1475-2875-8-265 PMC2787522

[15] Ameh J, Ahmad RM, Ekeh N, et al Laboratory diagnosis of malaria: Comparing giemsa stained thick blood films with rapid diagnostic test (RDT) in an endemic setting in North-west Nigeria. J Med Lab Diagn. 2012;3(2):10–15. https://doi.org/10.5897/JMLD12.001

[16] Wilson ML Malaria rapid diagnostic tests. Clin Infect Dis [serial online]. 2012;54(11):1637–1641. Available from: http://www.ncbi.nlm.nih.gov/pubmed/22550113 10.1093/cid/cis228 22550113

[17] Who TT, Malaria WHO, Product RDT, et al WHO Global Malaria Programme Information note on recommended selection criteria for procurement of malaria rapid diagnostic tests (RDTs). WHO Publ. 2012;64(December):1–13.

[18] Azikiwe CCA, Ifezulike CC, Siminialayi IM, et al A comparative laboratory diagnosis of malaria: Microscopy versus rapid diagnostic test kits. Asian Pac J Trop Biomed [serial online]. 2012;2(4):307–310. Available from: http://www.sciencedirect.com/science/article/pii/S222116911260029X 10.1016/S2221-1691(12)60029-X PMC3609291 23569920

[19] Falade CO, Adesina-Adewole B, Dada-Adegbola HO, et al Evaluation of Paracheck-Pf(TM) rapid malaria diagnostic test for the diagnosis of malaria among HIV-positive patients in Ibadan, south-western Nigeria. Pathog Glob Health [serial online]. 2013;107(2):69–77. Available from: http://www.pubmedcentral.nih.gov/articlerender.fcgi?artid=4001481&tool=pmcentrez&rendertype=abstract 10.1179/2047773213Y.0000000077 PMC4001481 23683333

[20] Mills LA, Kagaayi J, Shott JP, et al Performance of a prototype malaria rapid diagnostic test versus thick film microscopy among HIV-positive subjects in rural Rakai, Uganda. Trans R Soc Trop Med Hyg. 2010;104(3):237–239. https://doi.org/10.1016/j.trstmh.2009.07.030 19765788 10.1016/j.trstmh.2009.07.030 PMC2928654

[21] Andreoli A, Giorgetti PF, Pietra V, et al Evaluation of a PfHRP-2 based rapid diagnostic test versus microscopy method among HIV-positive and unknown serology patients in Ouagadougou, Burkina Faso. Am J Trop Med Hyg. 2015;92(4):834–837. https://doi.org/10.4269/ajtmh.13-0757 25667051 10.4269/ajtmh.13-0757 PMC4385783

[22] Jegede FE, Oyeyi TI, Abdulrahman SA, et al Effect of HIV and malaria parasites co-infection on immune-hematological profiles among patients attending anti-retroviral treatment (ART) clinic in Infectious Disease Hospital Kano, Nigeria. PLoS One. 2017;12(3):e0174233 https://doi.org/10.1371/journal.pone.0174233 28346490 10.1371/journal.pone.0174233 PMC5367709

[23] World Health Organization Methods manual for Malaria microscopy, method identification and qualitfication of malaria in thick and thin blood film. 2015 Geneva: WHO.

[24] Smart LR, Orgenes N, Mazigo HD, et al Malaria and HIV among pediatric inpatients in two Tanzanian referral hospitals: A prospective study. Acta Trop. 2016;159:36–43. https://doi.org/10.1016/j.actatropica.2016.03.019 27001145 10.1016/j.actatropica.2016.03.019 PMC4867247

[25] Chinkhumba J, Nyanda M, Skarbinski J, et al Performance of two malaria rapid diagnostic tests in febrile adult patients with and without human immunodeficiency virus-1 infection in Blantyre, Malawi. Am J Trop Med Hyg. 2012;86(2):199–202. https://doi.org/10.4269/ajtmh.2012.11-0350 22302848 10.4269/ajtmh.2012.11-0350 PMC3269267

[26] Lee N, Gatton ML, Pelecanos A, et al Identification of optimal epitopes for Plasmodium falciparum rapid diagnostic tests that target histidine-rich proteins 2 and 3. J Clin Microbiol. 2012;50(4):1397–1405. https://doi.org/10.1128/JCM.06533-11 22259210 10.1128/JCM.06533-11 PMC3318543

[27] Baker J, McCarthy J, Gatton M, et al Genetic diversity of Plasmodium falciparum histidine-rich protein 2 (PfHRP2) and its effect on the performance of PfHRP2-based rapid diagnostic tests. J Infect Dis [serial online]. 2005;192:870–877. Available from: http://www.ncbi.nlm.nih.gov/pubmed/16088837 10.1086/432010 16088837

[28] Murillo Solano C, Akinyi Okoth S, Abdallah JF, et al Deletion of Plasmodium falciparum Histidine-Rich Protein 2 (pfhrp2) and Histidine-Rich Protein 3 (pfhrp3) Genes in Colombian Parasites. PLoS One [serial online]. 2015;10(7):e0131576 Available from: http://www.ncbi.nlm.nih.gov/pubmed/26151448%5Cnhttp://www.pubmedcentral.nih.gov/articlerender.fcgi?artid=PMC4494814 10.1371/journal.pone.0131576 PMC4494814 26151448

[29] Deme AB, Park DJ, Bei AK, et al Analysis of pfhrp2 genetic diversity in Senegal and implications for use of rapid diagnostic tests. Malar J [serial online]. 2014;13(1):34 Available from: http://www.pubmedcentral.nih.gov/articlerender.fcgi?artid=3913323&tool=pmcentrez&rendertype=abstract 10.1186/1475-2875-13-34 PMC3913323 24472178

[30] Wurtz N, Fall B, Bui K, et al Pfhrp2 and pfhrp3 polymorphisms in Plasmodium falciparum isolates from Dakar, Senegal: Impact on rapid malaria diagnostic tests. Malar J [serial online]. 2013;12:34 Available from: http://www.pubmedcentral.nih.gov/articlerender.fcgi?artid=3571878&tool=pmcentrez&rendertype=abstract 10.1186/1475-2875-12-34 PMC3571878 23347727

[31] Abdallah JF, Okoth S, Fontecha GA, et al Prevalence of pfhrp2 and pfhrp3 gene deletions in Puerto Lempira, Honduras. Malar J [serial online]. 2015;14(1):19 Available from: http://www.malariajournal.com/content/14/1/19 10.1186/s12936-014-0537-7 PMC4308922 25604310

[32] Kumar N, Singh JP, Pande V, et al Genetic variation in histidine rich proteins among Indian Plasmodium falciparum population: Possible cause of variable sensitivity of malaria rapid diagnostic tests. Malar J. 2012;11(1):298 https://doi.org/10.1186/1475-2875-11-298 22929537 10.1186/1475-2875-11-298 PMC3475030

[33] Tseroni M, Pervanidou D, Tserkezou P, et al Field application of SD bioline malaria Ag Pf/pan rapid diagnostic test for malaria in Greece. PLoS One. 2015;10(3):1–11. https://doi.org/10.1371/journal.pone.0120367 10.1371/journal.pone.0120367 PMC4372373 25803815

[34] Koita OA, Doumbo OK, Ouattara A, et al False-negative rapid diagnostic tests for malaria and deletion of the histidine-rich repeat region of the hrp2 gene. Am J Trop Med Hyg. 2012;86(2):194–198. https://doi.org/10.4269/ajtmh.2012.10-0665 22302847 10.4269/ajtmh.2012.10-0665 PMC3269266

[35] Kozycki CT, Umulisa N, Rulisa S, et al False-negative malaria rapid diagnostic tests in Rwanda: Impact of Plasmodium falciparum isolates lacking hrp2 and declining malaria transmission. Malar J. 2017;16:123 https://doi.org/10.1186/s12936-017-1768-1 28320390 10.1186/s12936-017-1768-1 PMC5359811

[36] Tinto H, Sombié O, Valea I, et al Field evaluation of SD Bioline Malaria Antigen P. f ® for Plasmodium falciparum malaria diagnosis in Nanoro, Burkina Faso. Afr J Parasitol Res. 2015;4(11):161–165.

[37] Tiono AB, Diarra A, Sanon S, et al Low specificity of a malaria rapid diagnostic test during an integrated community case management trial. Infect Dis Ther [serial online]. 2013;2(1):27–36. Available from: http://www.ncbi.nlm.nih.gov/pubmed/25135821 10.1007/s40121-013-0006-6 PMC4108095 25135821

[38] van den Broek I, Hill O, Gordillo F, et al Evaluation of three rapid tests for diagnosis of P. falciparum and P. vivax malaria in Colombia. Am J Trop Med Hyg. 2006;75(6):1209–1215. https://doi.org/75/6/1209 [pii] 17172395

[39] Rakotonirina H, Barnadas C, Raherijafy R, et al Accuracy and reliability of malaria diagnostic techniques for guiding febrile outpatient treatment in malaria-endemic countries. Am J Trop Med Hyg. 2008;78(2):217–221. 18256418

[40] Bharti PK, Silawat N, Singh PP, et al The usefulness of a new rapid diagnostic test, the First Response(®) Malaria Combo (pLDH/HRP2) card test, for malaria diagnosis in the forested belt of central India. Malar J [serial online]. 2008 [cited 2018 Jul 11];7:126 Available from: http://www.pubmedcentral.nih.gov/articlerender.fcgi?artid=2478667&tool=pmcentrez&rendertype=abstract 10.1186/1475-2875-7-126 PMC2478667 18620560

[41] Mtove G, Nadjm B, Amos B, et al Use of an HRP2-based rapid diagnostic test to guide treatment of children admitted to hospital in a malaria-endemic area of north-east Tanzania. Trop Med Int Health. 2011;16(5):545–550. https://doi.org/10.1111/j.1365-3156.2011.02737.x 21320243 10.1111/j.1365-3156.2011.02737.x PMC3469736

[42] Gillet P, Mori M, Van Esbroeck M, et al Assessment of the prozone effect in malaria rapid diagnostic tests. Malar J [serial online]. 2009;8:271 Available from: http://www.pubmedcentral.nih.gov/articlerender.fcgi?artid=2789093&tool=pmcentrez&rendertype=abstract 10.1186/1475-2875-8-271 PMC2789093 19948018

[43] Kamya MR, Gasasira AF, Achan J, et al Effects of trimethoprim-sulfamethoxazole and insecticide-treated bednets on malaria among HIV-infected Ugandan children. AIDS. 2007;21(15):2059–2066. https://doi.org/10.1097/QAD.0b013e3282ef6da1 17885296 10.1097/QAD.0b013e3282ef6da1

[44] Mishra LC, Bhattacharya A, Sharma M, et al Short report: HIV protease inhibitors, indinavir or nelfinavir, augment antimalarial action of artemisinin in vitro. Am J Trop Med Hyg. 2010;82(1):148–150. https://doi.org/10.4269/ajtmh.2010.09-0427 20065012 10.4269/ajtmh.2010.09-0427 PMC2803526

[45] Andrews KT, Fairlie DP, Madala PK, et al Potencies of human immunodeficiency virus protease inhibitors in vitro against Plasmodium falciparum and in vivo against murine malaria. Antimicrob Agents Chemother. 2006;50(2):639–648. https://doi.org/10.1128/AAC.50.2.639-648.2006 16436721 10.1128/AAC.50.2.639-648.2006 PMC1366900

[46] Parikh S, Gut J, Istvan E, et al Antimalarial activity of human immunodeficiency virus type 1 protease inhibitors. Antimicrob Agents Chemother [serial online]. 2005;49(7):2983–2985. Available from: http://www.ncbi.nlm.nih.gov/pubmed/15980379%5C; http://www.ncbi.nlm.nih.gov/pmc/articles/PMC1168637/pdf/0090-05.pdf 10.1128/AAC.49.7.2983-2985.2005 PMC1168637 15980379

[47] Kattenberg JH, Ochodo EA, Boer KR, et al Systematic review and meta-analysis: Rapid diagnostic tests versus placental histology, microscopy and PCR for malaria in pregnant women. Malar J. 2011;10(1):321 https://doi.org/10.1186/1475-2875-10-321 22035448 10.1186/1475-2875-10-321 PMC3228868

[48] World Health Organization Making it work informal consultation on field trials and quality assurance on malaria rapid diagnostic tests regional office for the Western Pacific [homepage on the Internet]. Vol. 5 WHO; 2003 Available from: http://www.who.int/malaria/publications/atoz/rdt2.pdf

